# Text-Book of Gynæcology

**Published:** 1888-11

**Authors:** 


A TEXT-BOOK OF GYNECOLOGY, designed for the student and general practitioner. By Professor A. C. Cowperthwaite, M. D., Ph. D., LL. D. Chicago: Gross & Delbridge. 1888.

It must require considerable courage in any physician to write a new work upon "Gynecology," as the field has been so thoroughly gleaned over and over again. Upon receiving this volume of Professor Cowperthwaite's we hoped for something new and strong, but, alas! we have the same old routine of anatomy, of diagnosis, of descriptions of instruments, tables, and pos tions. As to the treatment, it is chiefly local; little reliance seems to be placed upon internal medication, yet it has been demonstrated in many cases that nearly all of the diseases of women *can be cured* by internal medication. It is idle to claim that this view of their treatment is only he d by "theorists," for it is not true. We cannot agree with Professor Cowperthwaite in his views upon treatment in general. Therapeutic indications are given in many cases, and in others remedies likely to be called for are named. The work is well edited and published, it is concise and clear in style, and will be of service to medical students.

